# Xeno-Free Propagation of Spermatogonial Stem Cells from Infant Boys

**DOI:** 10.3390/ijms20215390

**Published:** 2019-10-29

**Authors:** Lihua Dong, Murat Gul, Simone Hildorf, Susanne Elisabeth Pors, Stine Gry Kristensen, Eva R. Hoffmann, Dina Cortes, Jorgen Thorup, Claus Yding Andersen

**Affiliations:** 1 Laboratory of Reproductive Biology, Rigshospitalet, University Hospital of Copenhagen, 2100 Copenhagen, Denmark; murat.guel@regionh.dk (M.G.); susanne.elisabeth.pors@regionh.dk (S.E.P.); Stine.Gry.Kristensen@regionh.dk (S.G.K.); claus.yding.andersen@regionh.dk (C.Y.A.); 2 Department of Urology, Aksaray University School of Medicine, Aksaray 68100, Turkey; 3 Department of Pediatric Surgery, Rigshospitalet, Copenhagen University Hospital, 2100 Copenhagen, Denmark; simonehildorf@gmail.com (S.H.); joergen.mogens.thorup@regionh.dk (J.T.); 4 Center for Chromosome Stability, Institute of Molecular and Cellular Medicine, 2200 Copenhagen, Denmark; eva@sund.ku.dk; 5 Faculty of Health and Medical Sciences, University of Copenhagen, 2200 Copenhagen, Denmark; Dina.Cortes@regionh.dk; 6 Department of Pediatrics, Hvidovre, Copenhagen University Hospital, 2650 Copenhagen, Denmark

**Keywords:** Spermatogonial stem cells, xeno-free culture, male fertility cryopreservation, xeno-transplantation, in vitro propagation

## Abstract

Spermatogonial stem cell (SSC) transplantation therapy is a promising strategy to renew spermatogenesis for prepubertal boys whose fertility is compromised. However, propagation of SSCs is required due to a limited number of SSCs in cryopreserved testicular tissue. This propagation must be done under xeno-free conditions for clinical application. SSCs were propagated from infant testicular tissue (7 mg and 10 mg) from two boys under xeno-free conditions using human platelet lysate and nutrient source. We verified SSC-like cell clusters (SSCLCs) by quantitative real-time polymerase chain reaction (PCR) and immune-reaction assay using the SSC markers undifferentiated embryonic cell transcription factor 1 (UTF1), ubiquitin carboxyl-terminal hydrolase isozyme L1 (UCHL1), GDNF receptor alpha-1 (GFRα-1) Fα and promyelocytic leukaemia zinc finger protein (PLZF). The functionality of the propagated SSCs was investigated by pre-labelling using green fluorescent Cell Linker PKH67 and xeno-transplantation of the SSCLCs into busulfan-treated, therefore sterile, immunodeficient mice. SSC-like cell clusters (SSCLCs) appeared after 2 weeks in primary passage. The SSCLCs were SSC-like as the UTF1, UCHL1, GFRα1 and PLZF were all positive. After 2.5 months’ culture period, a total of 13 million cells from one sample were harvested for xenotransplantation. Labelled human propagated SSCs were identified and verified in mouse seminiferous tubules at 3–6 weeks, confirming that the transplanted cells contain SSCLCs. The present xeno-free clinical culture protocol allows propagation of SSCs from infant boys.

## 1. Introduction

Advances in cancer detection and therapy have greatly improved survival rates for childhood cancer patients and nowadays the vast majority will become long-term survivors. Approximately 1 in 530 young adults between the ages of 20 and 39 years is a childhood cancer survivor [1]. However, treatment of childhood cancers can adversely impact on the reproductive function [2]. To restore fertility in childhood cancer survivors, options for fertility preservation prior to gonadotoxic cancer therapies are needed [3]. For adult men, semen cryopreservation has been well-established for many years. However, cryopreservation of spermatozoa is not an option for prepubertal boys as they have no spermatogenesis yet [4]. Cryopreservation of immature testicular biopsy (ITT) for storage of spermatogonial stem cells (SSCs) is a preventive strategy for prepubertal boys facing gonadotoxic cancer therapy which has been available for more than a decade in different reproductive centres worldwide [5,6]. Furthermore, a number of non-malignant diseases may cause infertility, where patients also potentially could benefit from testicular tissue cryopreservation [5]. For instance, about 35% of boys who undergo orchidopexy, might exhibit reduced sperm concentrations, although early orchiopexy is suggested to limit the risks [7]. Nowadays, about 17% of azoospermia men have a history of cryptorchidism [8].

Testicular tissue engraftment, SSC auto-transplantation and in vitro spermatogenesis are considered as the three main approaches to rescue fertility after the boys have reached adulthood [9]. However, all strategies are still experimental. Establishment of a standard clinical procedure for cryopreserved testicular tissue to rescue fertility is an urgent demand.

SSC transplantation to restore spermatogenesis has been reported in mice first [10,11], and subsequently demonstrated in other mammalian animals, such as pig, cow, and monkey [12,13,14]. SSC auto-transplantation is now being proposed as a strategy to reinitiate spermatogenesis and rescue male infertility in the clinic [15]. To implement SSC transplantation methods successfully, in vitro propagation of SSCs is an essential step due to the limited number of SSCs in cryopreserved tissue [16]. In addition, the malignant cells may be eliminated and eventually disappear during the process of SSC propagation [17].

Currently, there are more than 20 published reports about human SSC culture [18]. Each centre has a unique protocol to culture human SSCs and used different outcome assessments, therefore, no consensus is accepted so far to replicate an SSC culturing method. Noticeably, in all studies, animal-derived products such as bovine serum albumin (BSA) or fetal bovine serum (FBS) were used in the culture media. Xenogeneic culture carries risks, in particular of transmission of known or as yet unrecognized xenogeneic infectious agents from animals to human beings and from recipients of xenogeneic transplants to their contacts and the public at large. Since xeno-free serum replacement has not been introduced so far, the clinical utilization of the SSCs is not possible. Therefore, establishing a xeno-free protocol for human SSC culture has utmost importance for clinical translation.

Although an announcement reported the establishment of a xeno-free culture system for expansion of human SSC, the culture media contained FBS and thereby xenogeneic [19]. In this paper, we present a xeno-free culture system using clinical-grade human platelet lysate (hPL) for the integration of SSC propagation into clinical application. The expanded SSCs were verified by xenotransplantation in sterile nude mice.

## 2. Results

### 2.1. Quantification of Germ Cells in Testicular Biopsies

**Figure 1:**
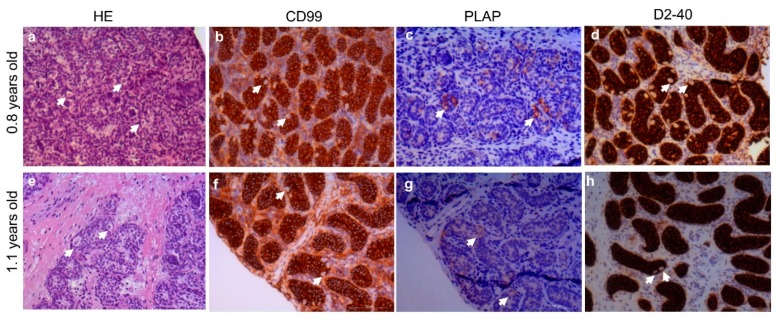
Hematoxylin and eosin (HE) staining and immunohistochemistry by CD99, PLAP and D2-40 in undescended testis aged 0.8 and 1.1 years old, respectively. HE staining (**a**,**e**); CD99 immunohistochemistry (**b**,**f**); PLAP immunohistochemistry (**c**,**g**); D2-40 immunohistochemistry (**d**,**h**); Arrows show the germ cells. CD99: cluster of differentiation 99; PLAP: placental alkaline phosphatase, D2-40: podoplanin; Scale bars: 100 µm.

To explore the potential SSC expansion in vitro, two testis samples from boys with bilateral cryptorchidism were investigated. The germ cell numbers per seminiferous tubule cross-section were 0.035 and 0.444, respectively. The number of type A dark spermatogonia (A_d_ spermatogonia) per seminiferous tubule cross-section was 0.004 and 0.01, respectively. The histological appearance and immunohistochemical analysis are shown in Figure 1.

### 2.2. Expansion of Spermatogonial Stem Cells (SSCs) In Vitro

**Figure 2:**
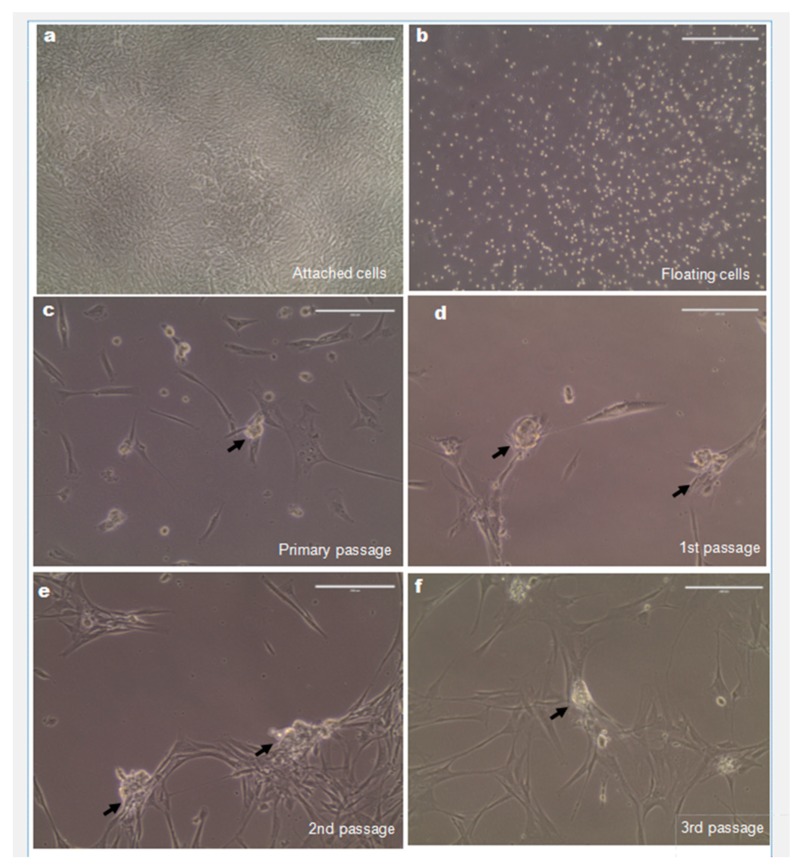
Spermatogonial stem cell (SSC)-like cell clusters were continuously formed in vitro. (**a**) The attached cell from the first plate formed confluence after 12 days. (**b**) SSC-enriched fractions (floating cell in first plates) was subsequently cultured in the StemPro-34 stem cell xeno-free media. (**c**–**f**) SSC-like cell clusters were formed in primary, first, second and third passages derived from SSC-enriched fractions cultured in xeno-free media. Arrows show the SSC-like cell clusters. Scale bars: 200 µm.

To propagate SSCs in vitro, a differential plate method was applied. After overnight incubation, floating cells considered as SSC enriched were isolated and cultured in StemPro-34 medium completed with multiple growth factors and hPL (Figure 2b). The attached cells in minimum essential media (MEM) media, considered as somatic cells, were confluent after 12 days and no clusters formed (Figure 2a). After 2 weeks, SSC-like cell clusters formed and developed as the grape-like cell clusters (Figure 2c). After around 1 month, the cells were passaged. Clusters that consistently formed in passage one, two and three are shown in Figure 2d–f. After 2.5 months’ culture period, a total of 13 million cells from one sample were harvested for xenotransplantation.

### 2.3. Molecular Analysis of SSC-Like Cells

**Figure 3:**
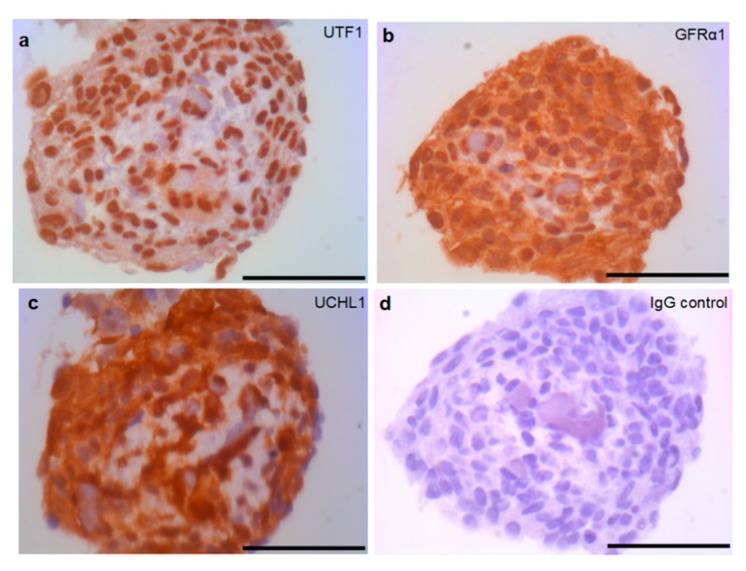
SSC-like cell clusters express SSC markers. Immunostaining of cross sections of SSC-like cell clusters by (**a**) undifferentiated embryonic cell transcription factor 1 (UTF1), (**b**) GDNF family receptor alpha-1 (GFRα-1), (**c**) ubiquitin carboxyl-terminal hydrolase isozyme L1 (UCHL1), and (**d**) IgG negative control. Scale bars: 50 µm.

**Figure 4:**
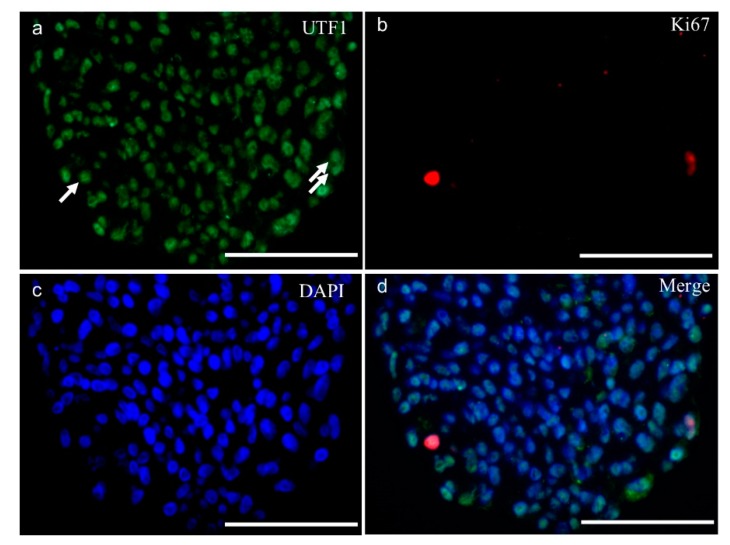
SSC-like cell clusters contain a few proliferating cells. Double immunofluorescence staining of SSC-like cell clusters (**a**) undifferentiated embryonic cell transcription factor 1 (UTF1); (**b**) antigen KI-67 (Ki67); (**c**): nuclear staining with 4′,6-diamidino-2-phenylindole (DAPI); (**d**) merged from three channels. Arrows showing the proliferating cells in the surface layer of clusters. Scale bars: 50 µm.

**Figure 5:**
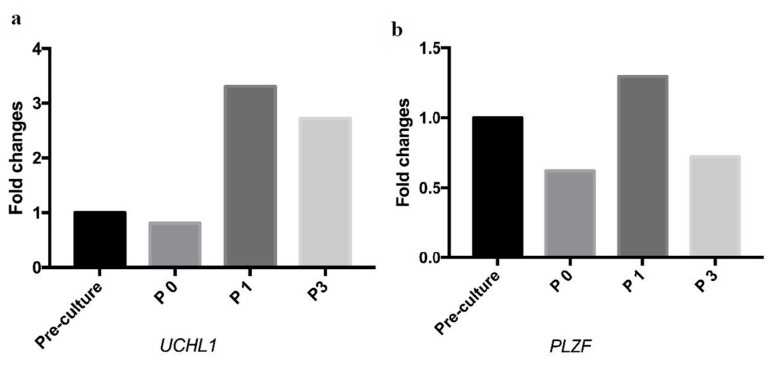
SSC-like cell clusters transcribe the message RNA of SSC markers during passaging. (**a**) Real-time quantification of the expression level of the SSC markers, ubiquitin carboxyl-terminal hydrolase isozyme L1 (UCHL1) and (**b**) promyelocytic leukaemia zinc finger protein (PLZF) during passaging. Pre-culture: the digested testicular cells. P0: Primary passage. P1: First passage. P3: Third passage.

To investigate the molecular properties of SSC-like cells a sectioned SSC-like cell cluster was immunohistochemically stained using different SSC markers, including undifferentiated embryonic cell transcription factor 1 (UTF1), GDNF family receptor alpha-1 (GFRα-1), ubiquitin carboxyl-terminal hydrolase isozyme L1 (UCHL1), which are all positively shown in SSC-like cell clusters (Figure 3). The antibody specificity was demonstrated by immunohistochemistry on a 0.8 years old cryptorchid testis (supplementary Figure S1). The proliferation rate was investigated using Ki67 antibody. Dual immunostaining with UFT1 and Ki67 is shown in Figure 4, which demonstrated few proliferating cells in the clusters. The SSC features of SSC-like cell clusters (SSCLCs) was further confirmed via real-time quantitative polymerase chain reaction (PCR) using different probes, including SSC markers, UCHL1 and promyelocytic leukaemia zinc finger protein (PLZF) from single cell cluster of different passages (Figure 5).

### 2.4. Sterilization of Nude Mice by Intratesticular Busulfan Injection

**Figure 6:**
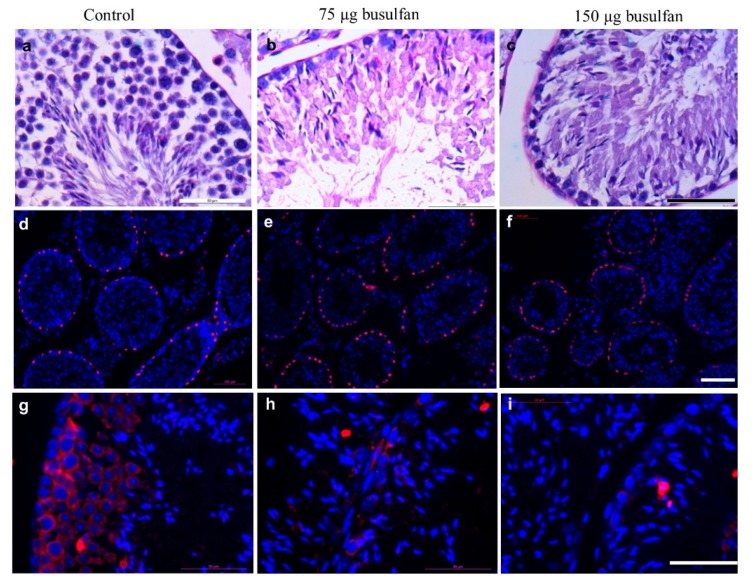
Testis from nude mice sterilized by intratesticular busulfan injection. **a**–**c**: Cross sectional histological analysis of mouse testis by periodic acid–Schiff (PAS) staining. (**a**) nontreated control (**b**) two weeks after intratesticular busulfan injection with either 75 µg or (**c**) 150 µg. Scale bar: 50 µm. **d**–**f**: Cross sectional immunofluorescent analysis of mouse testis by Sertoli cell marker SRY-Box 9 (SOX9; red) and DNA visualization by DAPI staining (blue) (**d**) non-treated control (**e**) two weeks after intratesticular busulfan injection with either 75 µg or (**f)** 150 µg. Scale bar: 100 µm. **g**–**i**: Cross sectional immunofluorescent analysis of mouse testis by germ cell marker VASA (red) and DNA visualization by DAPI staining (blue) (**g**) non-treated control (**h**) two weeks after intratesticular busulfan injection with either 75 µg or (**i**) 150 µg. Scale bar: 50 µm.

To eliminate endogenous germ cells for SSC transplantation, nude mice were sterilized using intratesticular injection with either 75 or 150 µg busulfan. The efficiency of the intratesticular injection of busulfan was evaluated by sacrificing two mice two weeks after injection. Histological evaluation demonstrated that both 75 and 150 µg of busulfan resulted in no spermatogenesis compared to the multiple cell layers found in the non-treated control group (Figure 6a–c). The endogenous germ cell devastation was confirmed by immunostaining with Sertoli cell marker SOX9 and germ cell marker VASA (Figure 6d–i). We observed that 75 µg busulfan was sufficient to deplete the endogenous germ cells, therefore, the recipient mice were prepared by intra-testicular injection with 75 µg of busulfan.

### 2.5. The SSC-Like Cells Settle in the SSC Niche after Xenotransplantation

**Figure 7:**
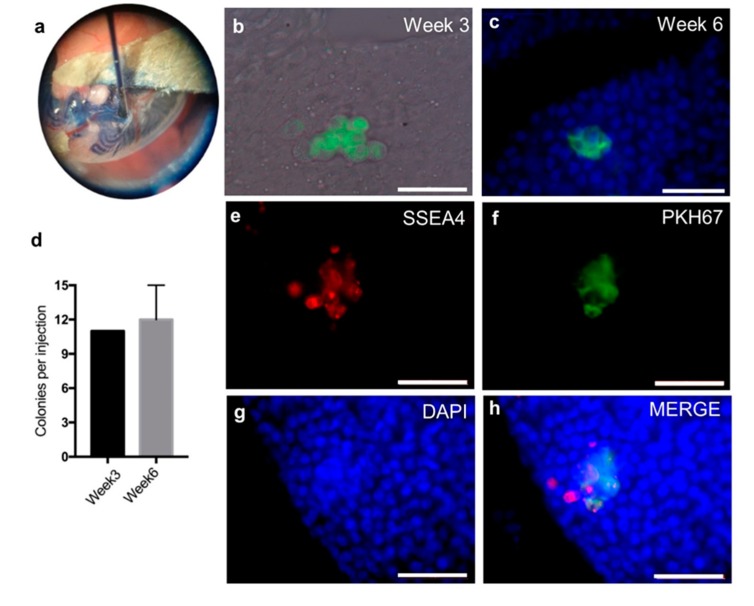
Xenotransplantation of the human cells into busulfan treated nude mice. (**a**) Demonstration of successful transplantation of human SSC-like cells into murine seminiferous tubules using trypan blue. (**b**) PKH67 (a green fluorescent cell linker) pre-labelled cells forming colonies in recipient testis merged with bright channel three weeks after transplantation. (**c**) PKH67 pre-labelled cells forming colonies in recipient testis merged with DAPI staining six weeks after transplantation. (**d**) Mean number with standard deviation of colonies formed numbers in recipient mouse testis. Three weeks after transplantation (*n* = 1) and six weeks after transplantation (*n* = 3). **e–h**: Whole mount immunofluorescent analysis of mouse testis six weeks after transplantation by SSC marker, stage-specific embryonic antigen-4 (SSEA4; red) and DNA visualization by DAPI staining (blue) (**e**) SSEA4; (**f**) PKH67; (**g**) DAPI and (**h**) merged pictures from previous three channels. Scale bar: 50 µm.

To verify SSC identity after propagation, xenotransplantation was performed. A total number of 13 million cells were collected after 2.5 months propagation with 3 passages and pre-labelled with green fluorescent cell linker PKH67. Around one million labelled cells were transplanted into seminiferous tubules of each testis via the efferent duct (Figure 7a). The colonies of PKH67 labelled cells in seminiferous tubules of the recipient mice were identified after 3 and 6 weeks (Figure 7b,c). The number of colonies was quantified as around 11 colonies (more than four cells in one cluster) with per million transplanted cells after three and six weeks (Figure 7d). Whole-mount testicular immunostaining showed that the SSC marker stage-specific embryonic antigen-4 (SSEA4) was expressed in PKH67 positive colonies (Figure 7e–h), indicating the successful migration of donor cells onto the basement membrane of seminiferous tubules in recipients.

## 3. Discussion

Establishment of a xeno-free culture condition to propagate human SSCs is a key step to advance SSC transplantation to restore the spermatogenesis to the clinic level. Here we for the first time cultured SSCs from infant boys using hPL and human serum albumin instead of xenogeneic factors and used culture conditions compatible with clinical conditions. A total of 13 million cells from one sample was harvested after three passages. Positive immunostainings and qPCR analysis of SSCLCs using different SSC markers demonstrated the presence of SSCs. Furthermore, the functional properties of the propagated cells were validated by the positive recovery of SSCs which were transplanted into the seminiferous tubules of immunodeficient mice after a grafting period of either three or six weeks.

This study demonstrates that human SSCs can be propagated under strict xeno-free conditions to maintain functional and molecular characteristics of SSCs. Several other groups demonstrated the possibility to cultivate human SSCs colonies from adult testis biopsies in co-culture with Sertoli cells or on feeder-free conditions [20,21], or even after purification of SSC sorted via SSEA-4 or GPR125 expression [22,23]. Recently, we reported the propagation of human SSC-like cells from infant boys under xenogeneic culture conditions [24]. However, the media used in these studies included animal-derived products such as BSA, FBS or other xenogeneic factors, which preclude clinical utilization of the SSCs [25,26,27,28]. We investigated a clinically acceptable human substitution, which is already used in a clinical setting, by replacing FBS with 2% hPL and replacing BSA with human serum albumin. Therefore, the culture conditions described in the present study advances human SSCs transplantation to the clinical level.

The starting material for our studies consisted with an average of around 8.5 mg testicular tissue per sample in which histological examinations showed an average of 0.25 germ cells per seminiferous tubule cross-section. This number of cells is lower than that in healthy boys at a similar age [29,30,31], showing impairment of germ cell development in undescended testis [31,32]. The number of germ cells per seminiferous tubule (age-matched) observed in prepubertal testicular tissue with a malignant disease resembles or is slightly higher than those we observed in boys with bilateral cryptorchidism [33], indicating that the methods developed in this study may have wider applications also in boys with childhood cancer receiving potential gonadotoxic treatments.

SSCs were isolated by the differential plating method to enrich human SSCs under xenogeneic conditions [22,23]. In our study, SSCLCs formed as grape-like cell clusters attached the fibroblast-like cells after around 2 weeks. Individual big round cells were identifiable in the cell clusters compared to the condensed SSC clusters using FBS media [34,35]. The different morphology may result from a different exposure to hormones and growth factors in hPL and FBS. The morphological difference of mesenchymal stem cells was also observed in hPL versus FBS media. While more elongated spindle-shaped stem cells were found in hPL media, more flat cells were observed in FBS media [36].

We first validated the propagated SSCLCs evaluating putative SSC markers, UTF1, GFRα-1, and UCHL1. UTF1 is a transcription repressor located in the nucleus regulating embryonic stem-cell proliferation and differentiation [37] and plays a key role for the maintenance of SSC by the mediation of the phosphatase and tensin homolog (PTEN) signaling pathway in mice [38]. In human testis, UTF1 is restricted to expression in spermatogonia [39,40]. In addition, Persio et al. [41] did not observe the ki67 positive cells in UTF1 expressing spermatogonia in adult testis. We found UTF1 only localized in the nucleus of SSCLCs and very few UTF1 positive cells showed proliferation marker, indicating the most cell became quiesced in SSCLCs while only a few cells are proliferative active. GDNF secreted by testicular somatic cells is required for SSC homeostasis [42]. GFRα-1, a human spermatogonial marker, is a GDNF family receptor that mediates the GDNF-induced autophosphorylation and activation of the RET receptor for SSC self-renewal [43,44]. GFRα-1 was found to be expressed in SSCLCs of the present study, thereby further demonstrating the presence of SSCs in the clusters. Recently, testicular cells were intensively characterized using single-cell RNA-Seq approaches, which identified the subset populations of spermatogonia [45,46,47]. During the SSC proliferation and differentiation, primitive spermatogonia have GFRα-1^low^ UTF1^high^ cell population and differentiate into GFRα-1^high^ UTF1^low^ cell population based on pseudotime cell trajectory analysis of the sequencing data [48]. However, Persio and co-workers investigated adult human testis mainly based on whole-mount immunostaining approaches and suggested that the beginning of the spermatogenic lineage expresses a high level of GFRα-1, then UTF1 subsequently becomes expressed and GFRα-1 expression decreases [41]. Despite the controversial suggestions for the temporal expression during spermatogonial differentiation in adult human testis, both subpopulations of spermatogonia are considered as part of the human SSC pool. The SSCLCs contained UCHL1 positive cells which represent spermatogonia in adult testis [49].

We and other studies [34,35,50] have determined the cell identity of propagated human SSCs initially based on the expression of a group of putative SSC markers. However, recent studies suggested that several putative SSC markers, such as PLZF, GFRα-1, UCHL1, and ITGA6 are also expressed in testicular somatic cells [27,51]. To exclude debatable conclusion based on previous markers, we performed immunostaining of an additional SSC marker UTF1, which was intensively used to determine human SSCs in culture especially in the combination of SSEA4 [18].

To obtain additional evidence for the SSC nature of the propagated cells, we performed a functional assay by transplantation of the propagated human cells to busulfan-treated immunodeficient mice. Several xenogeneic SSC transplantations were successfully performed using busulfan-treated immunodeficient male mice as recipients from different donor mammalian species, such as marmosets, macaques, and humans [52,53,54]. The donor-derived germ cell colonies from all non-rodent mammals were found in the basal lamina of mouse seminiferous tubules. However, the differentiation of donor-derived SSCs was not observed in the recipient testis, probably due to the species-specific differentiation factors in examined mammals [55]. Sadri-Ardekani and co-workers [34,35] were the first to introduce xenotransplantation of propagated human cells into sterile nude mice. The authors presented a single donor cell in mouse seminiferous tubules, which was not further characterized. Mohaqiq et al. [56] observed spermatogenesis from transplanted human SSCs in mouse seminiferous tubules after in vitro xenotransplantation which has different conditions compared to nude mice in vivo xenotransplantation. In the present study, the donor-derived colonies with more than four cells were identified in the recipient mouse testis three and six weeks after transplantation. Xenotransplantation of freshly isolated human SSCs also formed human cell-derived colonies in seminiferous tubules of recipient mice [28,57]. In general, the donor cell derived-colonies formation in immunocompromised mice testis are considered to represent SSCs [58]. In addition, we investigated the cell identity by whole-mount staining of recipient mouse testis using an SSC marker SSEA4 [59]. Almost all PHK67 pre-labelled transplanted cell shows the SSEA4 signals, indicating the transplanted human cells still keep the SSC activity. A few single SSEA4 positive cells which were near to the PHK67 labelled colony probably represent the remaining mouse endogenous germ cells after busulfan treatment. The co-localization of mouse and human SSCs showed that a healthy mouse SSC niche also supported the formation of human cells colonies. However, a definitive conclusion on whether human SSCs are the colony-forming cells in the recipient mouse testis is lacking, and further functional assays need to be developed to evaluate the self-renewal and differentiation capacity of human SSCs.

There are several limitations to our study. First, the SSCs could not be obtained purely as they also contain some fibroblast-like cells. Moreover, the proliferation rate of SSCs could not be calculated precisely since we did not quantify the population proportion of SSCs in culture by fluorescence-activated flow cytometry. In addition, we performed the experiments from freshly collected biopsy samples which may be different from freeze-thaw biopsies. Moreover, the composition of hPL we used is not well defined and the key factors for the survival and proliferation of human SSC in vitro need to be determined. Last but not least, the functionality of human cell-derived colonies in recipient mice is awaiting further investigations.

## 4. Materials and Methods

### 4.1. Testicular Biopsies

Testicular biopsies samples were obtained from 2 patients (0.8 and 1.1 years old) undergoing an operation for bilateral cryptorchidism and scheduled for cryopreservation. None of these boys had previously received chemotherapy, radiotherapy or hormonal treatment. The present study was approved by the ethical committee of the Capital Region of Copenhagen (H-2-2012-060, 11 June 2012). In order to analyze the germ cells, testis tissue samples were fixed in Stieve’s solution overnight at room temperature, embedded in paraffin, and sectioned at 2µm. The histological sections were stained with hematoxylin and eosin (HE), and immunohistochemical staining including, cluster of differentiation 99 (CD99) (1:100, 12E7, Dako), Glostrup, Denmark, podoplanin (D2-40) (1:25, M3619, Dako) and placental-like alkaline phosphatase (PLAP) (1:200, PL8-F6, Biogenex, Fremont, CA, USA). The number of germ cells per tubular transverse section (G/T) and the number of Ad spermatogonia per tubular transverse section were measured from HE stained sections as well as immunohistochemical staining of CD99, D2-40, and PLAP [60]. For each patient, at least 100 cross-sectional tubules were examined.

### 4.2. Testicular Cell Isolation, Culture, and Cryopreservation

Fresh testicular biopsies weighing 7 and 10 mg were enzymatically digested by two steps, firstly incubating in 450 U/mL collagenase type I (Worthington), 450 U/mL Hyaluronidase type II (Sigma-Aldrich, Søborg, Denmark), 500 U/mL Trypsin TRL3 (Worthington) and 8 µg/ml DNase for 15 min followed by incubating in 450 U/mL collagenase type I, 450 U/mL Hyaluronidase type II and 8 µg/ml DNase for 30 min, to prepare a cell suspension. Testicular cells were collected and cultured overnight in uncoated dishes in supplemented alpha-modified MEM media with 4-(2-hydroxyethyl)-1-piperazineethanesulfonic acid (HEPES), penicillin-streptomycin (100 μg/mL) (Invitrogen), containing 10% hPL and 0.1% heparin (1000 IE/ml, Amgros, Denmark) at 35 °C and 5% CO2. After overnight incubation, floating cells were collected and cultured in uncoated dishes with supplemented StemPro-34 (Gibco, Life Technologies, Nærum, Denmark). In addition, 0.5% penicillin-streptomycin, recombinant human EGF (20 ng/mL, Sigma-Aldrich), recombinant human basic FGF (10 ng/mL, Sigma-Aldrich) recombinant human GDNF (10 ng/mL, Sigma-Aldrich), recombinant human LIF (10 ng/mL, EMD Millipore Corp), 2% hPL (Lot3, Rigshospitalet) and and 0.1% heparin were added. The cells were cultured in Nunclon delta surface plates (Thermo Fisher Scientific, Nærum, Denmark) at 35 °C in a humidified atmosphere with 5% CO_2_. Cells were passaged with Accutase (Invitrogen) every 2–4 weeks to new dishes depending on the confluence of testicular cells in dish and all cells were passaged three times.

### 4.3. Immunostaining

Testicular tissues or SSCLCs were embedded in alginate followed by 4% agarose, fixed in 4% paraformaldehyde for around 16 h and 4 h, respectively, embedded in paraffin, and cut into 5 µm sections. The sections were deparaffinized, subjected to antigen retrieval treatment using TEG buffer (10 mM Tris, 0.5 mM ethylene glycol-bis(β-aminoethyl ether)-*N*,*N*,*N*′,*N*′-tetraacetic acid (EGTA), pH 9), and blocked for 0.5 h at room temperature in 1% BSA in Tris-buffered saline (TBS) buffer (50 mM Tris, 150 mM NaCl, pH 7.6) before primary antibodies were applied. The following antibodies were used: a mouse monoclonal anti-UTF1 antibody (1:50; Mab4337, Merck Millipore, Darmstadt, Germany), a polyclonal rabbit anti- GFRα-1 antibody (1:50; ab84106, Abcam, Cambridge, UK), a mouse monoclonal anti-UCHL1 (1:100, sc-271639, Santa Cruz, Heidelberg, Germany), a polyclonal rabbit anti-Ki67 antibody (1:100; ab15580, Abcam), a polyclonal rabbit anti-SOX9 antibody (1:100; ab5535, Merck Millipore), a polyclonal goat anti-human VASA antibody (1:100; AF2030, Novus biologicals, Oxford, UK), and universal negative control serum (NC498H, Biocare Medical, Copenhagen, Denmark). The slides were washed 3 times. For immune-fluorescence staining, the sections were stained with an Alexa Fluor 594 donkey anti-goat IgG antibody (1:500 Jackson Immunoresearch, Skanderborg, Denmark) or a FITC-conjugated donkey anti-mouse IgG antibody (1:500; Jackson Immunoresearch) or Alexa Fluor 594 donkey anti-rabbit IgG antibody (1:500 Jackson Immunoresearch) for 1 h at room temperature. The DNA was visualized using DAPI staining before mounting slides with ProLong Gold Antifade Mountant (Life Technology). For immunohistochemical staining, sections were merged in 1.5% H_2_O_2_ in TBS buffer to quench endogenous peroxidase before applying blocking buffer. Signals were visualized on the section by incubation with the secondary antibodies, either rabbit anti-mouse-HRP or goat anti-rabbit-HRP (1:100, Dako, Glostrup, Denmark) for 10 min at room temperature, visualized by peroxidase reaction with 3,3′-diaminobenzidine tetrahydrochloride (Dako) for 1–2 min, and counterstained with hematoxylin. Sections were analyzed with a Leica Microsystems. Pictures were taken with a Leica digital camera.

### 4.4. Gene Expression Analysis

Total RNA from cell clusters and dissolved single-cell suspensions were extracted with the Absolutely RNA Nanoprep Kit (Agilent Technologies) according to the manufacturer’s instructions. cDNA synthesis was performed with High Capacity cDNA Reverse Transcription Kit (Applied Biosystems) according to the manufacturer’s instructions. The gene expression was detected on the LightCycler 480 Instrument II (Roche Diagnostics). The reaction mix consisted of 2 µL of template cDNA, 5 µL of Taqman universal PCR master mix (Applied Biosystems), 0.5 µL of Taqman primer assays specific for for glyceraldehyde 3-phosphate dehydrogenase (GAPDH; Hs025978991_g1, Thermo Fisher Scientific), for PLZF (Hs00957433_m1, Thermo Fisher Scientific), and for UCHL1 (Hs00985157_m1, Thermo Fisher Scientific) and 2.5 µL of H_2_O to a final volume of 10 µL. A 96-well plate was used, and each sample was run in duplicates. The PCR cycling conditions were set to one initiation cycle at 95 °C for 10 minutes, followed by 45 cycles of 95 °C for 10 seconds, 60 °C for 15 seconds, 70 °C for 10 seconds, and finally, one cycle at 95 °C for 1 minute. The raw Ct value was reported from the Roche RCR software.

### 4.5. Xenotransplantation

The use of animals was approved by the Animal Experiments Inspectorate (case file 2015-15-0201-00505) under Danish legislation. Six nude mice homozygous for *Foxn1* mutation (Taconic, Borup, Denmark) were used. Mice were fed pellets and water ad libitum and kept under controlled 12/12 h light/dark cycles at 20–22 °C. Mice were anaesthetized by use of zoletil (Virbac, Nice, France), xylazin (Scanvet, Fredensborg, Denmark) and butorphanol (Zoetis, Parsippany, NJ, USA). Postoperative analgesia was provided by the use of buprenorphine (ReckittBenckiser, England, UK) and carprofen (Norbrook, England, UK). Mice were single-housed up until euthanasia, which was done by cervical dislocation [61]. When the mice were 5 weeks old each testis was injected with the doses of 75 or 150 μg busulfan (Sigma) dissolved in dimethyl sulfoxide (DMSO) and delivered in a total volume of 20 μl through two different injection sites [62]. The sterilization efficiency was investigated by PAS and immunostaining by sacrificing two mice after two weeks and SSC xenotransplantation was performed according to the protocol [63]. Around one million of propagated cells in a volume of 15 μl was injected through an efferent duct. The injection was visualized by the addition 0.04% Trypan Blue. The propagated cells were pre-labelled by PHK67 (Sigma) according to manufacturer instructions [64].

### 4.6. Whole-Mount Immunofluorescence Staining of Seminiferous Tubules

The protocol of whole-mount immunofluorescence staining of seminiferous tubules was performed with slight modification according to the literature [65]. Briefly, tunica was removed from recovered mouse testis and the whole testis enzymatically digested with Collagenase IV (Gibco, Life Technologies) and DNase I (Sigma) followed by fixation with 4% formaldehyde (Invitrogen). Seminiferous tubules were stained with primary antibodies, mouse monoclonal IgG3 anti-SSEA4 (1:50, 60062, STEMCELL, Cambridge, UK), secondary antibodies with Alexa Fluor 568 donkey anti-mouse IgG antibody (1:500, Jackson immunoresearch). The DNA was visualized using DAPI staining before mounting slides with ProLong Gold Antifade Mountant (Life Technology). Samples were analyzed with a Leica Microsystems. A Leica digital camera was used for analysis and image capture.

## 5. Conclusions

We succeeded in the long-term culture of human SSCLCs starting from infant boys under xeno-free conditions. In the biopsy specimens, one of the patients had quite a few germ cells, and even in that case we managed to achieve many SSCLCs. The presence of specific SSC markers expressed in SSCLCs in culture and the human cell-derived colonies formed in recipient mice by xenotransplantation demonstrated the SSCLC activity. The results are encouraging for a continued research effort to develop a clinically acceptable solution for maintaining fertility in young boys in need of preserving fertility.

## Supplementary Materials

Supplementary materials can be found at https://www.mdpi.com/1422-0067/20/21/5390/s1.

## Abbreviations

BSA	Bovine Serum Albumin
EGF	Epidermal growth factor
FBS	Fetal Bovine Serum
FGF2	Basic fibroblast growth factor
GDNF	Glial cell line-derived neurotrophic factor
GFRα1	GDNF family receptor alpha-1
hPL	Human platelet lysate
MEM	Minimum Essential Medium
ITGA6	Integrin alpha 6
ITT	Immature testicular testis
LIF	Leukaemia inhibitory factor
PLZF PTEN	Promyelocytic leukaemia zinc finger protein Phosphatase and tensin homolog
SSEA4	Stage-specific embryonic antigen-4
SSC	Spermatogonial stem cell
SSCLC	Spermatogonial stem cell-like cells
UCHL1	Ubiquitin carboxyl-terminal hydrolase isozyme L1
UTF1	Undifferentiated embryonic cell transcription factor 1
