# Supplementary material for: Xeno-Free Propagation of Spermatogonial Stem Cells from Infant Boys
Source: Int J Mol Sci. 2019 Oct 29;20(21):5390. doi: 10.3390/ijms20215390 (PMC6862004; doi:10.3390/ijms20215390)
Supplement: Supplementary file 1 [file ijms-20-05390-s001.pdf]

Supplemental data

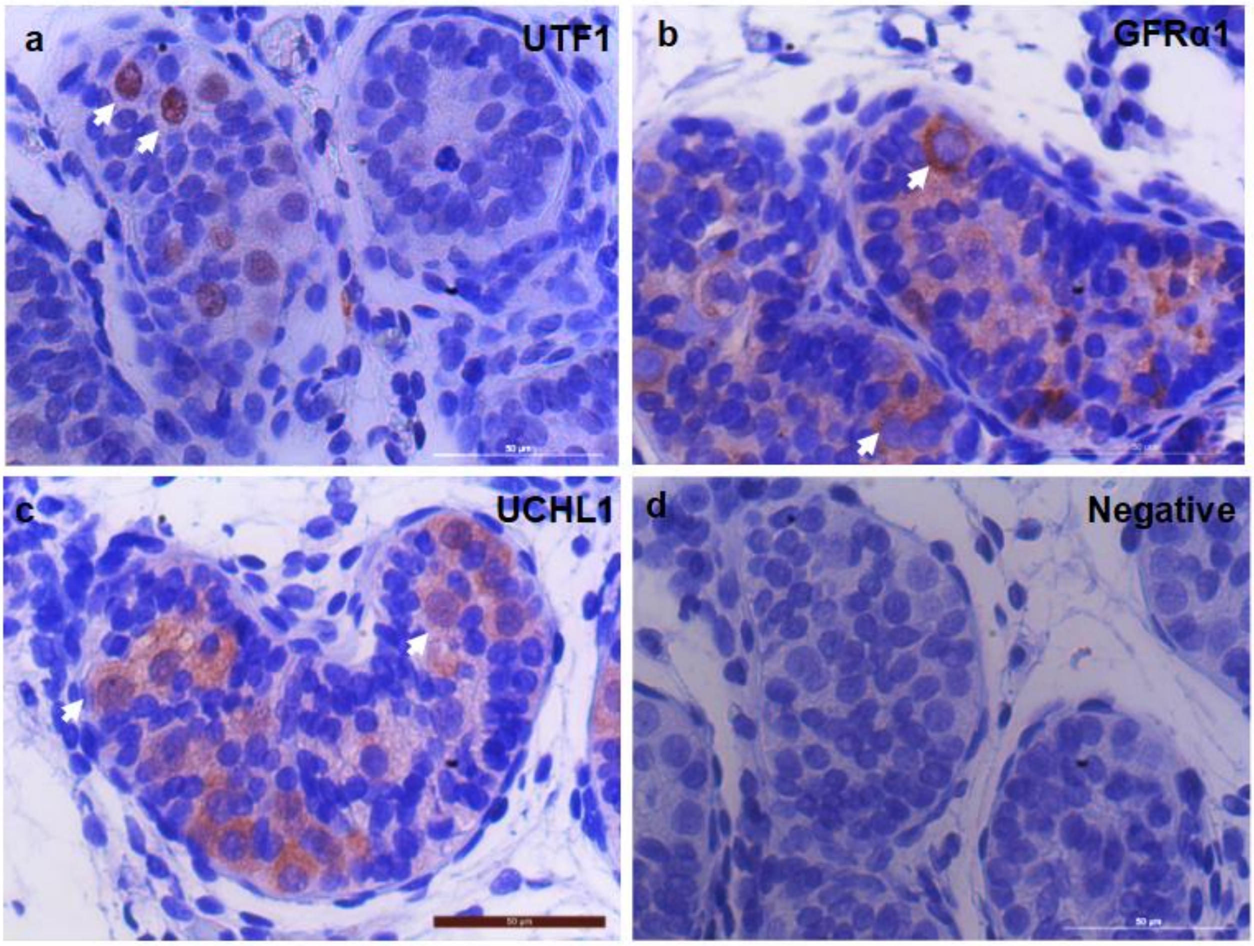

Supplemental figure S1 Immunohistochemical staining analysis of undescended testis (0.8 years old). a: undifferentiated embryonic cell transcription factor 1 (UTF1), b: GDNF family receptor alpha-1 (GFRα1), c: ubiquitin carboxyl-terminal hydrolase isozyme L1 (UCHL1). Arrows show germ cells with positive signals, d: IgG negative control. Scale bars: 50 μm.
